# A Cognitive Model of Alcohol Use Among Taiwanese Adolescents: The Influence of Alcohol Expectancies and Drinking Refusal Self-Efficacy

**DOI:** 10.3390/healthcare13222981

**Published:** 2025-11-20

**Authors:** Mei-Yu Yeh, Chyi-In Wu, Yen-Hua Shih, Yu-Kuei Chen

**Affiliations:** 1 Department of Nursing, Kang Ning University, Taipei 114, Taiwan; yehdiana8@gmail.com; 2 Institute of Sociology, Academia Sinica, Taipei 115, Taiwan; sss1ciw@gate.sinica.edu.tw; 3 Department of Nursing, National Tai Nen Junior College of Nursing, Tainan 700, Taiwan; yasshih@ntin.edu.tw; 4 Department of Nutrition, I-Shou University, Kaohsiung 840, Taiwan

**Keywords:** adolescents, drinking expectancy questionnaire, drinking refusal self-efficacy

## Abstract

Background: Drinking alcohol of adolescents is an important issue in Taiwan. The purpose of the research is to determine how drinking expectancy and drinking refusal self-efficacy influence drinking behavior among Taiwanese adolescents based on a cognitive model of alcohol consumption. Methods: In this cross-sectional study, a total of 908 students, selected from 10th to 12th grade of six high schools in Taiwan, were stratified randomly. Pearson correlation, and multiple regression analyses were conducted to examine the relationships among drinking expectancy, refusal self-efficacy, and alcohol use, including drinking frequency and drunkenness frequency. Results: There was significant positive relationship between drinking expectancies, and drinking and drunkenness frequency; and negative correlation between drinking refusal self-efficacy, and drinking and drunkenness frequency. Multiple regression analysis revealed that (1) tension reduction, sexual enhancement, social pressure, emotional relief, and opportunity to drink significant predicted drinking frequency (Adjusted R^2^ = 0.352, *p* < 0.001) and (2) tension reduction, increased confidence, cognitive enhancement, and social pressure significant predicted drunkenness frequency (Adjusted R^2^ = 0.226, *p* < 0.001). Conclusions: Adolescents have positive outcome expectancies regarding increased drinking frequency. Under the pressure of social interaction, drinking was the most difficult to refuse. Alcohol expectancy and drinking refusal self-efficacy have both shown notable influences on Taiwanese adolescents.

## 1. Introduction

Alcohol is one of the most used substances among adolescents [1]. Adolescent alcohol consumption can harm emotional, cognitive, and psychosocial development [1] and increase the risk of problematic drinking, with these harms potentially persisting into adulthood [2]. Alcohol-related morbidity and mortality also contribute significantly to the global public health burden [3]. According to the World Health Organization (WHO), alcohol is the fifth leading risk factor for the global burden of disease, with one person dying every minute from alcohol-related harm. The WHO recommends that nations prioritize addressing the issue of problematic drinking [4]. Consequently, the health and social problems caused by alcohol consumption have garnered considerable attention from the global medical community [5,6].

A recent study published in *The Lancet Psychiatry* reported a strong association between early, long-term heavy drinking in adolescence and the onset of depression in late adolescence [7]. Increased frequency and quantity of alcohol consumption among adolescents significantly elevate the risk of suicidal ideation and suicide attempts [8,9]. These findings highlight a strong correlation between adolescents’ suicidal ideation, suicide attempts, and the developmental trajectories of their long-term drinking behaviors. Alcohol consumption likely plays a key precipitating role in the emergence of suicidal ideation or intentions among adolescents [10]. Notably, the negative consequences associated with alcohol consumption are critical predictors of suicide attempts in this population [11].

Liou & Chou (2001) conducted an epidemiological survey in Taiwan from 1991 to 1996 and found that the prevalence rate of adolescent drinking behavior (defined as drinking at least once per month) was 16.7%, with 22.9% for males and 10.1% for females [12]. Age-stratified analysis revealed a prevalence rate of 11.3% among 12-year-olds, rising to 31.4% among 17-year-olds. This increasing prevalence in early adolescent drinking behaviors in Taiwan closely mirrors the developmental trajectories observed in North American and European adolescents [13]. This early research suggests a growing crisis in adolescent drinking behavior in Taiwan.

Although Taiwan’s legal framework explicitly prohibits the sale of alcohol to individuals under 18 years old, the prevalence of alcohol consumption among adolescents aged 12 to 17 has steadily risen over the past decade. Reports from Taiwan indicate that the prevalence rate of alcohol use among this demographic has doubled in the past 10 years [14]. The 2009 National Health Interview and Drug Abuse Survey reported prevalence rates of 5.6% among adolescents aged 12–14 and 11.6% among those aged 15–17. By 2018, these rates had increased to 12.9% and 28.3%, respectively, suggesting that nearly 500,000 adolescents in Taiwan had developed a habit of alcohol consumption [14].

Given that adolescents are still in critical stages of physiological and psychological development, alcohol use increases their risk of social maladaptation and behavioral problems, including school dropout, substance abuse, and traffic accidents. Moreover, the long-term developmental and health consequences, such as alcohol addiction, unemployment, and criminal behavior, cannot be ignored [15]. The factors influencing adolescents who drink behavior are diverse and complex. Previous studies have shown a close relationship between adolescents who drink behavior and two key constructs: drinking expectancies and drinking refusal self-efficacy [16]. Neighbors et al. (2019) emphasized that these constructs are critical in understanding the cognitive processes underlying drinking behavior [17]. These constructs highlight the significance of individual, environmental, outcome expectancies, and efficacy expectancies factors in shaping behavior. When individuals hold positive expectations about drinking, such as its ability to reduce social pressure, alleviate tension, and enhance social relationships, these expectations significantly increase the likelihood of drinking behavior. However, adolescents who believe that drinking reduces stress are more likely to increase their alcohol consumption [16]. Consequently, the more positive the drinking expectancies among adolescents, the higher their drinking frequency and quantity [18,19,20].

Although drinking behavior has been studied in Taiwanese adolescents, the relationship between drinking expectancies and drinking refusal self-efficacy and behavior has not. Therefore, this study aims to investigate the influence of drinking expectancies and drinking refusal self-efficacy on adolescent drinking behavior with goal of developing intervention strategies to reduce the concurrent and potential long-term negative effects of alcohol consumption on adolescents as they transition into adulthood.

Based on the cognitive model of drinking [16], we hypothesized that positive alcohol expectancies would be positively associated with drinking frequency and drunkenness, and drinking refusal self-efficacy would be negatively associated with these behaviors.

## 2. Materials and Methods

### 2.1. Participants

This study employed a cross-sectional design and utilized stratified random sampling to select six high schools in Taiwan, with two schools randomly selected from each of the northern, central, and southern regions. Students from grades 10 to 12 were selected, with 60 students randomly selected from each grade, totaling 1080 students. Data collection was conducted using a questionnaire survey, with students self-reporting their responses. A total of 1042 questionnaires were collected, with 134 incomplete or missing responses excluded, resulting in 908 valid questionnaires (87.1%). A minimum sample of 138 was required to detect a medium effect size (f^2^ = 0.15) with power = 0.95 and α = 0.05 using G Power 3.1 version. The final sample of 908 well exceeded this threshold, ensuring adequate statistical power.

### 2.2. Measures

#### 2.2.1. Alcohol Consumption

This study referenced the definitions of adolescent drinking behavior proposed by Yeh and Chiang, which include two dimensions: (1) the frequency of alcohol consumption in the past year and (2) the frequency of drunkenness in the past year [21]. Both were measured on a six-point scale: 1 point for never drink, 2 points for drinking rarely (less than once per month on average), 3 points for occasionally drink (1–2 times per month on average), 4 points for often drink (3–4 times per month on average), 5 points for regularly (2–3 times per week on average), 6 points for almost daily drink (4 or more times per week on average). Negative alcohol-related consequences, such as missing school, drunk driving, or encountering trouble due to drinking, were measured with three additional questions [21].

#### 2.2.2. Drinking Expectancy Questionnaire (DEQ)

The Drinking Expectancy Questionnaire (DEQ) includes five subscales: negative consequences (16 items), increased confidence (12 items), sexual enhancement (3 items), cognitive enhancement (3 items), and tension reduction (3 items), a total of 37 items. Responses are rated on a five-point Likert scale [22]. The negative consequences items (e.g., Drinking alcohol makes me tense or Drinking makes me bad-tempered) assess the awareness of negative outcomes, with higher scores indicating stronger negative expectancies. Positive expectancy subscales included increased confidence (e.g., Drinking makes me feel outgoing and friendly), sexual enhancement (e.g., Drinking makes me more sexually responsive), cognitive enhancement (e.g., I feel restless when drinking alcohol), and tension reduction (e.g., When I am anxious or tense, I do feel a need for alcohol). Higher scores on these subscales indicate stronger positive alcohol expectancies.

Confirmatory factor analysis of the DEQ has demonstrated to have a stable factor structure (Goodness of fit index = 0.97) [22], with internal consistency reliability (Cronbach’s α) of 0.85 [23]. In this study, the DEQ exhibited high internal consistency (α = 0.87), with subscale reliabilities ranging from 0.72 to 0.84.

#### 2.2.3. Drinking Refusal Self-Efficacy Questionnaire-Revised (DRSE-R)

The Drinking Refusal Self-Efficacy Questionnaire-Revised (DRSE-R), developed by Oei, Hasking and Young (2005) [24], consists of 19 items across three subscales: social pressure (5 items), emotional relief (7 items), and opportunity to drink (7 items). Examples include When my friends are drinking (social pressure), When I feel sad (emotional relief), and When I am on the way home from school (opportunity to drink). Responses are rated on a six-point Likert scale, from 1 (definitely cannot refuse) to 6 (definitely can refuse). The DRSE-R has demonstrated strong internal consistency (Cronbach’s α = 0.87–0.94) and test–retest reliability (0.84–0.93). In this study, the DRSE-R exhibited high internal consistency (α = 0.95), with subscale reliabilities ranging from 0.93 to 0.98 [24].

### 2.3. Procedure

The questionnaires were translated into Chinese through a two-stage translation process and underwent reliability and validity testing before use. After receiving approval from the Human and Social Science Research Ethics Committee (No. AS-IRB-HS02-24002), we randomly selected schools and obtained consent from school administrators. Students were informed about the anonymous and voluntary nature of the study and were assured that there were no right or wrong answers, as the primary goal was to understand adolescent drinking behavior and its influencing factors. Parental consent was obtained, and students provided written informed consent before completing the questionnaire.

### 2.4. Data Analysis

Descriptive statistics were used to examine the demographic variables and frequency distribution of drinking behavior among participants. Pearson correlation and multiple regression analyses were conducted to explore the relationships and impacts of drinking expectancies and drinking refusal self-efficacy on alcohol consumption and intoxication frequency among Taiwanese adolescents. Prior to regression analysis, multicollinearity was assessed using Variance Inflation Factor (VIF) and Conditional Index (CI), with results showing VIF values below 5 and CI values below 30, indicating acceptable levels of multicollinearity. All predictors were entered simultaneously in multiple regression analyses using the enter method. Assumptions of linearity, homoscedasticity, and normality of residuals were verified. Multicollinearity diagnostics indicated acceptable VIF (<5) and Condition Index (<30) values. All statistical operations were performed using SPSS 25.0 for Windows. A *p*-value of 0.05 or lower was considered significant.

## 3. Results

This study included 908 adolescents aged 15 to 17 years, with a mean age of 17.06 years (SD = 0.87). Among the participants, 70.5% (640/908) were male, and 29.5% (268/908) were female. Regarding family structure, 75.6% (687/908) of participants lived with both parents, while 24.3% (221/908) came from single-parent families.

### 3.1. Drinking Behavior

As shown in Table 1, 37.6% (341/908) of adolescents reported no alcohol consumption in the past year. Meanwhile, 30.2% (274/908) drank rarely, and 32.2% (293/908) reported consuming alcohol at least one to two times per month. Regarding the frequency of becoming drunk, 68.1% (620/908) reported no instances of being drunk in the past year, while 31.9% (288/908) had experienced drunkenness. Additionally, 44.7% (406/908) of adolescents reported purchasing alcohol themselves within the past year. Almost seven percent (6.9%) of adolescents indicated that they drank with friends almost daily in the past year. Approximately 20.4% of participants drank with their families at least once per month.

### 3.2. Pearson Correlation Analysis

Table 2 displays the Pearson correlation between adolescent drinking, drinking expectancies (DEQ), and drinking refusal self-efficacy (DRSE). A significant positive correlation was found between tension reduction and both drinking frequency (γ = 0.389) and frequency of becoming drunk or intoxicated (γ = 0.336). Conversely, DRSE social pressure was significantly negatively correlated with drinking frequency (γ = −0.560) and frequency of becoming drunk (γ = −0.434).

### 3.3. Multiple Regression Analysis

As seen in Table 3, multiple regression analysis revealed that within the cognitive model of adolescent drinking, both drinking expectancies (total scores) and drinking refusal self-efficacy (total scores) significantly influenced drinking frequency (Adjusted R^2^ = 0.292, *p* < 0.001) and frequency of becoming drunk (Adjusted R^2^ = 0.154, *p* < 0.001). These two constructs explained 29.2% of the variance in drinking frequency and 15.4% of the variance in frequency of becoming drunk.

Table 4 show the significant predictors of drinking frequency within the five dimensions of drinking expectancies. These included sexual enhancement (t = −3.377, *p* < 0.001) and tension reduction (t = 2.191, *p* < 0.05). The sexual enhancement expectancy negatively predicted drinking frequency (β = –0.14, *p* = 0.001), suggesting that adolescents endorsing this belief tended to drink less frequently. The significant predictors of drinking refusal self-efficacy included social pressure (t = −9.462, *p* < 0.001), emotional relief (t = 3.128, *p* < 0.01), and opportunity to drink (t = −4.258, *p* < 0.001), collectively explaining 35.2% of the variance.

The significant predictors for increased frequency of drunkenness included tension reduction (t = 6.370, *p* < 0.001), increased confidence (t = −2.336, *p* < 0.05), cognitive enhancement (t = −3.267, *p* < 0.001), and drinking refusal self-efficacy under social pressure (t = −8.071, *p* < 0.001), collectively explaining 22.6% of the variance.

## 4. Discussion

According to the 2021 report from the Health Promotion Administration, Ministry of Health and Welfare in Taiwan, 30.6% of high school students (aged 15 to 17) reported drinking alcohol in the past month, while 19.4% admitted to becoming drunk. In the current study, 32.2% of adolescents reported drinking at least once a month in the past year, and 17% reported being drunk. These findings agree with the results of the 2021 survey. The same report also highlighted that 76.3% of adolescents who drink consumed more than one drink per occasion, with 80.4% of males and 70.8% of females reporting such behaviors. This suggests that more than 70% of adolescents who drink in Taiwan lack awareness of moderate drinking practices, leading to uncontrolled alcohol consumption [14].

Despite Taiwan’s legal prohibition on the sale of alcohol to individuals under 18, this study found that 23.2% of adolescents aged 15 to 17 reported purchasing alcohol themselves in the past year. The current study also found that 6.9% of adolescents reported drinking with friends almost daily in the past year. As Rossow suggested, self-reports of drinking are often underreported, which may also be the case in this study [25]. This suggests that enforcement of the policy may be insufficient, allowing underage adolescents to purchase alcohol from stores, potentially contributing to the rising frequency and quantity of alcohol consumption among this demographic [15].

Whether parents should allow adolescents to drink alcohol remains a controversial issue. This study revealed that over 20% of adolescents who drank with family members at least once per month. Drinking at home or alcohol provided by parents is often rooted in the belief that it offers a safer drinking environment and establishes family-based drinking norms, which may serve as protective factors against problematic drinking [26,27]. However, other studies suggest that parental allowance of adolescents who drank at home can create permissive family norms, increasing the risk of higher drinking frequency, heavy alcohol use, and problematic behaviors [28,29,30].

### 4.1. Predictors of Adolescent Drinking Behavior

Multiple regression analysis revealed that drinking expectancies and drinking refusal self-efficacy were significant predictors of drinking and frequency of becoming drunk among adolescents in our cognitive model of alcohol use. Positive expectancies related to sexual enhancement and tension reduction were significant predictors of drinking frequency. Adolescents who drank to reduce tension exhibited higher drinking frequency, while those who expected alcohol to enhance sexual interest exhibited lower drinking frequency. Drinking refusal self-efficacy in the context of social pressure, emotional relief, and drinking opportunities also significantly predicted drinking frequency.

Significant predictors in frequency of becoming drunk included positive expectancies (increased confidence, cognitive enhancement, and tension reduction) as well as drinking refusal self-efficacy (social pressure). Among these, social pressure was identified as a critical factor influencing both drinking frequency and frequency of becoming drunk. Alcohol expectancies are a critical factor in the development of problematic alcohol use. Past research has shown that adolescents hold positive expectations about drinking, including enhanced social interaction, relaxation, reduced tension, increased confidence, and escapism from stress or negative emotions [18,31]. Drinking refusal self-efficacy is a protective factor. Thus, adolescents with impaired social skills could be more vulnerable to binge drinking. In this study, frequency of being drunk was not driven by increased confidence or cognitive enhancement but was instead associated with “it would be better drunk, escapism, or self-medication behaviors which may reduce stress and manage emotional distress [32,33,34].

### 4.2. Implications for Intervention

While drinking expectancies strongly predict adolescent drinking behavior [31], social and cultural differences in how these expectancies influence behavior remain [35]. For instance, studies have shown that adolescents with higher drinking refusal self-efficacy in social pressure contexts drink less frequently [23]. This study highlights the importance of refusal self-efficacy in shaping adolescent drinking behaviors across different groups. Since life stressors are linked to substance abuse and suicidal behaviors in adolescents, an inability to cope with such stressors may increase alcohol consumption and related health risks [6]. The comorbidity of heavy drinking and depressive symptoms may result from expectancies that alcohol reduces tension and stress [36].

The findings of this study underscore the need for monitoring adolescents who drink behaviors and implementing early psychosocial and behavioral interventions to mitigate harm. Parents play a crucial role in shaping adolescents’ views towards alcohol. Sellman et al. (2010) recommend that parents educate adolescents about the physical and mental impacts of alcohol, engage in open communication, and teach strategies to resist peer pressure [37]. Additionally, policies prohibiting underage alcohol purchase and consumption should be enforced more effectively, alongside public awareness campaigns to address parental and community perceptions of alcohol use.

### 4.3. Limitations

This study has several limitations. First, its cross-sectional design precludes causal inference between alcohol expectancies, refusal self-efficacy, and drinking behaviors. Second, self-reported data may be subject to recall or social desirability bias. Third, this empirical study demonstrates the specificity of the DEQ and DRSE constructs, findings are of considerable importance. Future study is recommended to validate the cognitive model of adolescent alcohol use through structural equation modeling.

## 5. Conclusions

Because this study employed a cross-sectional design, causal relationships cannot be inferred; future longitudinal research is warranted to confirm these cognitive pathways. This study suggests that effective strategies for preventing adolescent problematic drinking should be grounded in cognitive models of drinking behavior. These strategies include clarifying positive drinking expectancies, emphasizing negative consequences, strengthening refusal self-efficacy under social pressure, promoting confidence without alcohol, and enhancing stress management and emotional regulation skills [23,38].

## Figures and Tables

**Table 1:** Frequency of adolescent drinking-related experiences.

Question	Never n (%)	Rarely n (%)	Occasionally n (%)	Often n (%)	Regularly n (%)	Almost Daily n (%)	Mean (SD)
1. Frequency of drinking	341 (37.6)	274 (30.2)	199 (21.9)	54 (5.9)	29 (3.2)	11 (1.2)	2.11 (1.14)
2. Frequency of drunk	620 (68.1)	135 (14.9)	146 (16.1)	3 (0.3)	3 (0.3)	3 (0.3)	1.50 (0.81)
3. Frequency of purchasing alcohol by oneself	502 (55.3)	195 (21.5)	170 (18.7)	30 (3.3)	10 (1.1)	1 (0.1)	1.74 (0.96)
4. Frequency of drinking with friends	383 (42.2)	286 (31.5)	157 (17.3)	13 (1.4)	6 (0.7)	63 (6.9)	2.08 (1.35)
5. Frequency of drinking with family	522 (57.5)	201 (22.1)	140 (15.4)	39 (4.3)	5 (0.6)	1 (0.1)	1.69 (0.93)

Note: Rarely (less than once per month on average); Occasionally (1–2 times per month on average); Often (3–4 times per month on average); Regularly (2–3 times per week on average); Almost daily (4 or more times per week on average).

**Table 2:** Pearson’ correlation between drinking behavior, expectancies and drinking refusal self-efficacy.

Variables	Drinking Frequency	Drunkenness Frequency	Mean (SD)
DEQ (Drinking expectancies)	0.380 **	0.282 **	79.94 (25.06)
negative consequences	0.286 **	0.176 **	29.90 (11.84)
tension reduction	0.354 **	0.239 **	28.15 (11.30)
increased confidence	0.389 **	0.336 **	7.24 (3.20)
cognitive enhancement	0.280 **	0.151 **	6.56 (2.60)
sexual enhancement	0.175 **	0.110 **	5.43 (2.80)
DRSE (Drinking refusal self-efficacy)	−0.513 **	−0.371 **	85.79 (24.21)
social pressure	−0.560 **	−0.434 **	20.09 (7.13)
emotional relief	−0.440 **	−0.331 **	30.55 (10.70)
opportunity to drink	−0.429 **	−0.271 **	35.13 (8.66)

Note: ** *p* < 0.01.

**Table 3:** Multiple regression analysis of the cognitive model for DEQ and DRSE-R total scores.

Variables	Drinking Frequency			Drunkenness Frequency		
	β	t	p	β	t	p
Constant		15.347	***		12.584	***
DEQ (Drinking expectancies)	0.194	6.253	***	0.147	4.404	***
DRSE (Drinking refusal self-efficacy)	−0.429	−13.842	***	−0.306	−9.038	***
R	0.542			0.395		
R^2^	0.294			0.156		
Adjusted R^2^	0.292			0.154		
F	188.279 ***			83.489 ***		

Note: *** *p* < 0.001.

**Table 4:** Multiple regression analysis of cognitive model for each dimension.

Variables	Drinking Frequency			Drunkenness Frequency		
	β	t	*p*	β	t	*p*
Constant		18.732	***		14.468	***
DEQ (Drinking expectancies)						
negative consequences	0.073	1.414	n.s.	0.060	1.072	n.s.
tension reduction	0.118	2.191	*	0.374	6.370	***
increased confidence	0.096	1.652	n.s.	−0.149	−2.336	*
cognitive enhancement	0.025	0.572	n.s.	−0.156	−3.267	***
sexual enhancement	−0.140	−3.377	***	−0.002	−0.044	n.s.
DRSE (Drinking refusal self-efficacy)						
social pressure	−0.464	−9.462	***	−0.432	−8.071	***
emotional relief	0.159	3.128	**	0.087	1.568	n.s.
opportunity to drink	−0.197	−4.258	***	0.010	0.188	n.s.
R	0.598			0.482		
R^2^	0.357			0.233		
Adjusted R^2^	0.352			0.226		
F	62.519 ***			34.098 ***		

Note: * *p* < 0.05; ** *p* < 0.01; *** *p* < 0.001; n.s.: non-significant.

## Data Availability

The datasets generated and analyzed during the current study are available from the corresponding author on reasonable request. The data are not publicly available due to their containing information that could compromise the privacy of research participants.
